# Chromatin-Independent Interplay of NFATc1 and EZH2 in Pancreatic Cancer

**DOI:** 10.3390/cells10123463

**Published:** 2021-12-08

**Authors:** Shilpa Patil, Teresa Forster, Kristina Reutlinger, Waltraut Kopp, Lennart Versemann, Jessica Spitalieri, Jochen Gaedcke, Philipp Ströbel, Shiv K. Singh, Volker Ellenrieder, Albrecht Neesse, Elisabeth Hessmann

**Affiliations:** 1 Department of Gastroenterology, Gastrointestinal Oncology and Endocrinology, University Medical Center Goettingen, 37075 Goettingen, Germany; shilpapatil528@gmail.com (S.P.); kristina.reutlinger@med.uni-goettingen.de (K.R.); wkopp@med.uni-goettingen.de (W.K.); lennart.versemann@t-online.de (L.V.); jspitali@med.uni-goettingen.de (J.S.); shiv.singh@med.uni-goettingen.de (S.K.S.); volker.ellenrieder@med.uni-goettingen.de (V.E.); albrecht.neesse@med.uni-goettingen.de (A.N.); 2 Department of Gastroenterology, Endocrinology, Metabolism and Clinical Infectiology, Philipps University Marburg, 35043 Marburg, Germany; terry.forster@web.de; 3 Clinical Research Unit KFO5002, University Medical Center Goettingen, 37075 Goettingen, Germany; jochen.gaedcke@med.uni-goettingen.de (J.G.); philipp.stroebel@med.uni-goettingen.de (P.S.); 4 Department of General-, Visceral-, and Pediatric Surgery, University Medical Center Goettingen, 37075 Goettingen, Germany; 5 Institute of Pathology, University Medical Center Goettingen, 37075 Goettingen, Germany

**Keywords:** chromatin, EZH2, NFATc1, pancreatic cancer, posttranslational EZH2 modification

## Abstract

Background: The Nuclear Factor of Activated T-cells 1 (NFATc1) transcription factor and the methyltransferase Enhancer of Zeste Homolog 2 (EZH2) significantly contribute to the aggressive phenotype of pancreatic ductal adenocarcinoma (PDAC). Herein, we aimed at dissecting the mechanistic background of their interplay in PDAC progression. Methods: NFATc1 and EZH2 mRNA and protein expression and complex formation were determined in transgenic PDAC models and human PDAC specimens. NFATc1 binding on the *Ezh2* gene and the consequences of perturbed NFATc1 expression on *Ezh2* transcription were explored by Chromatin Immunoprecipitation (ChIP) and upon transgenic or siRNA-mediated interference with NFATc1 expression, respectively. Integrative analyses of RNA- and ChIP-seq data was performed to explore NFATc1-/EZH2-dependent gene signatures. Results: NFATc1 targets the *Ezh2* gene for transcriptional activation and biochemically interacts with the methyltransferase in murine and human PDAC. Surprisingly, our genome-wide binding and expression analyses do not link the protein complex to joint gene regulation. In contrast, our findings provide evidence for chromatin-independent functions of the NFATc1:EZH2 complex and reveal posttranslational EZH2 phosphorylation at serine 21 as a prerequisite for robust complex formation. Conclusion: Our findings disclose a previously unknown NFATc1-EZH2 axis operational in the pancreas and provide mechanistic insights into the conditions fostering NFATc1:EZH2 complex formation in PDAC.

## 1. Introduction

Pancreatic ductal adenocarcinoma (PDAC) remains a major challenge in cancer medicine. The overall five-year survival rate of less than 10% of patients has remained unchanged for almost 20 years and the majority of PDAC patients do not survive the first year upon diagnosis [1]. Major causes for the dismal outcome are the exceptionally aggressive tumor biology with early onset of metastasis and the remarkable resistance to conventional chemotherapy. Despite significant efforts in translational PDAC research and irrespective of the huge technical advances which enabled the disentanglement of the molecular underpinnings of PDAC in high resolution [2,3,4], we still lack an in-depth understanding of the complex interplay of the different oncogenic drivers determining the particularly aggressive phenotype of PDAC.

Besides oncogenic KRAS and its downstream kinases [4,5], transcription factors and epigenetic regulators individually and jointly orchestrate transcription programs permissive for PDAC progression [6,7,8,9,10]. One of the chromatin-regulatory proteins which is regularly overexpressed in several malignancies including PDAC is the histone methyltransferase Enhancer of Zeste Homolog 2 (EZH2) [11,12]. As the catalytic subunit of the polycomb repressor complex 2 (PRC2), EZH2 targets lysine 27 on histone 3 (H3K27me3) for trimethylation, thus silencing gene expression [11]. We recently described EZH2-dependent transcriptional repression of the *GATA6* gene as a key event in counteracting classical molecular PDAC subtype identity, thus linking EZH2 expression and activity to a highly aggressive and invasive PDAC phenotype [7]. In contrast to its obvious tumor-promoting activities in established PDAC, EZH2 protects acinar cells from malignant transformation [13,14]. However, oncogenic KRAS activation switches EZH2 from a suppressor to a promoter of pancreatic carcinogenesis [14]. Mechanistically, we found that EZH2-dependent promotion of pancreatic carcinogenesis in the context of mutant *KRAS* involves the transcriptional activation of the *NFATc1* gene [14], which encodes for different alternative splicing variants of the calcium-/calcineurin-responsive inflammatory transcription factor Nuclear Factor of Activated T-cells (NFATc1). Based on its overexpression in approximately 70% of human PDAC specimens [15], and given its pleiotropic impact on controlling PDAC proliferation, invasion, metastasis, and acquisition of stemness features [15,16,17,18,19], NFATc1 represents a pivotal driver of PDAC development and progression. Importantly, upon EZH2 depletion in PDAC models with EZH2-disentangled, constitutive NFATc1 activation strongly abrogated the tumor-promoting activities of the transcription factor, both in vitro and in vivo [7]. Hence, the histone methyltransferase critically contributes to NFATc1-driven oncogenic activities in PDAC. Given the functional implication of EZH2 in NFATc1-controlled processes in PDAC progression [7], we proposed the existence of an NFATc1-EZH2 axis which underlies the regulation of joint oncogenic programs in PDAC. 

Herein, we provide mechanistic insights into the transcriptional regulation of *EZH2* expression and reveal solid complex formation of the histone methyltransferase and NFATc1 in murine and human PDAC. Surprisingly, integration of genome-wide NFATc1 and EZH2 binding and expression data do not link NFATc1:EZH2 complex formation with joint target gene regulation. Instead, our findings point towards chromatin-independent functions of the protein complex, thus adding another layer of complexity to the dynamic interplay of tumor-promoting regulatory proteins in PDAC. 

## 2. Material and Methods

### 2.1. Mouse Strains and In Vivo Experiments

*Kras^G12D^;pdx1-Cre-*(*Kras^G12D^), Kras^G12D^;Nfatc1^fl/fl^;pdx1-Cre-* (*Kras^G12D^; Nfatc1^fl/fl^*), and *Kras^G12D^;TP53^R172H/+^;pdx1-Cre-* (*KPC*) mice have been previously described [5,16,20]. Genotyping of transgenic mouse strains was performed as described previously [16]. Mice were sacrificed either at indicated time points to capture defined stages of pancreatic cancer development and progression or upon reaching endpoint criteria. While one part of the harvested tissue was formalin-fixed and paraffin embedded (FFPE) for subsequent immunohistochemical analysis, the second part of the harvested tissue was shock frozen for RNA and protein extraction. Patient-Derived Xenograft (PDX) models were generated from resected PDAC specimen as described previously [7]. All procedures were conducted in accordance with the protocols approved by the Institutional Animal Care and Use committee at the University Medical Center Goettingen (33.9-42502-04-14/1633, 33.9-42502-04-14/1634; 33.9-42502-04-19/3085, 33.9-42502-04-17/2074).

### 2.2. Acinar Cell Extraction

Acinar cell extraction of *Kras^G12D^* and *Nfatc1^fl/fl^;Kras^G12D^* mice was performed as described previously [16]. In brief, the pancreas of a six-week old mice was dissected and incubated at 37 °C in a collagenase VIII-containing solution. The minced pancreas was passed through a 100-μm nylon filter. After repetitive washing, acini were harvested for subsequent RNA and protein isolation.

### 2.3. Cell Culture, Transfection, and Treatment

NKC-II and KPC cells were isolated from tumor baring *NKC (cnNFATc1;Kras^G12D^;pdx1-Cre*) and *KPC* mice, respectively, as described before [15,18] and cultured using Dulbecco’s Modifies Eagle’s Medium (DMEM, Life Technologies, Carlsbad, NM, USA) containing 4.5 g/L D glucose, L-Glutamine supplemented with 10% FCS (fetal calf serum) and 1% NEAA (non-essential amino acids). PANC1 cells were cultivated using DMEM containing 4.5 g/L D-Glucose, L-Glutamine supplemented with 10% FCS (fetal calf serum). As shown previously [17], 10% FCS is sufficient to induce NFATc1 translocation into the nucleus. Cells were placed in incubator at 37 °C and 5% (*v*/*v*) CO_2_ and were regularly evaluated for mycoplasma contamination. siRNAs targeting NFATc1 were purchased from Ambion, Carlsbad, CA, USA. Cells were transfected with siRNA for 48 h using silentfect (Biorad, Bio Rad Laboratories, Hercules, CA, USA) in 200 µL of serum free media. Myctag-EZH2 constructs were a kind gift from Myles Brown, Dana Faber Institute, Boston, MA, USA, and have been described previously [21]. Then, 10 µg of EZH2 plasmids were transiently transfected into NKC-II cells utilizing lipofectamine 2000 (Invitrogen, Carlsbad, CA, USA). 

### 2.4. RNA Extraction and Quantitative Realtime-PCR

For RNA isolation, cells were scraped in TRIzol (Ambion, Life Technologies, Carlsbad, CA, USA) and the total cellular RNA was isolated by phenol-chloroform method. For RNA extraction from pancreatic mouse tissue, tissue was homogenized in RT-lysis buffer and RNA was extracted utilizing the PeqGOLD Total RNA kit 200 (Peqlab, VWR International, Radnor, PA, USA). Then, 1 µg of RNA was reverse transcribed into cDNA using iScript cDNA synthesis kit (BioRad, 170-8891, Bio Rad Laboratories, Hercules, CA, USA). Quantitative Realtime-PCR (q-RT-PCR) was performed in triplicates as described before [19]. All mRNA expression was normalized to the level of the housekeeping gene *RPLP0* and further normalized to control. Supplementary Table S1 lists the primers utilized for qPCR analysis. 

### 2.5. Human PDAC Material and Microdissection of Epithelial Tumor Tissue

PDAC tissue for subsequent generation of PDX-models, immunohistochemistry and microdissection was derived from the Department of General, Visceral and Pediatrics Surgery and the Institute of Pathology at the University Medical Center Göttingen in accordance to the ethical regulations of the institutes (11/5/17). 

To explore *NFATc1*- and *EZH2* expression in epithelial tumor tissue from human and murine PDAC, manual microdissection was performed as described previously [22,23]. Briefly, FFPE tissue slices were deparaffinized and stained with Hematoxylin (Sigma-Aldrich, St. Louis, MO, USA). At 40-fold magnification, representative tumor areas were identified. Using a pointed surgical blade, dissection of tumor cells was carried out. Dissected tissue was transferred to a tube for subsequent RNA extraction using the Qiagen AllPrep RNA FFPE Kit (Qiagen, Hilden, Germany) according to the manufacturer’s instructions.

### 2.6. Protein Harvesting, Western Blot, and Immunoprecipitation

Protein isolation of pancreatic tissue was performed as described previously [16]. For protein isolation from PDAC and acinar cells, cells were harvested in whole-cell lysis (WCL) buffer supplemented with 1X complete™ protease inhibitor cocktail (Roche, 11 697 498001; 25X stock; La Roche, Basel, Switzerland) and additional protease inhibitors—PMSF, NaF, and NaO—as described before [19]. Protein concentration was measured using Bradford reagent (BioRad, Protein Assay Dye Reagent concentrate; Bio Rad Laboratories, Hercules, CA, USA), following the standard protocol. Western blotting was performed as described in [7], and using the primary and secondary antibodies listed in Supplementary Table S2. Bands were visualized in Intas ECL Chemocam Imager (Intas Science Imaging, Goettingen, Germany) using chemiluminoscence (Perkin Elmer, Waltham, MA, USA). For immunoprecipitation (IP) experiments in WCL, cells were collected and lysed in WCL buffer including the inhibitors mentioned above. For IP experiments in nuclear lysates, the cytoplasmic fraction was obtained using buffer A containing 10 mM HEPES pH 7.9, 10 mM KCl, 100 µM EGTA, 100 µM EDTA, and protease inhibitors. Upon centrifugation, the supernatant containing the cytoplasmic fraction was discarded and the pellet was resolved in buffer C for isolation of the nuclear fraction (20 mM HEPES pH 7.9, 400 mM NaCl, 1 mM EGTA, 1 mM EDTA, protease inhibitor). Then, 400–600 µg of whole-cell lysate and 100–200 µg of nuclear lysate were utilized for precipitation of NFATc1 (Santa Cruz sc-7294; Santa Cruz Biotechnology, Dallas, TX, USA), pSer21EZH2 (Bethyl IHC-00388; Bethyl, Montgomery, AL, USA), Myctag (Cell Signaling #2272S; Cell Signaling Technology, Danvers, MA, USA), EZH2 (Cell Signaling #5246; Cell Signaling Technology, Danvers, MA, USA), HA (Cell Signaling #2367; Cell Signaling Technology, Danvers, MA, USA), or IgG (Santa Cruz, sc-2025; Santa Cruz Biotechnology, Dallas, TX, USA and Millipore #12-370; MilliporeSigma, Burlington, VT, USA) overnight at 4 °C, respectively. Pre-cleared protein A/G agarose beads were added and incubated for 2 h with gentle shaking. The samples were washed with lysis buffer and Phosphate-Buffered-Saline (PBS), before the complexes were resolved with 2× SDS and transferred to SDS-PAGE and Western blotting as described above. For IP experiments performed upon DNA fragmentation, nuclear cell lysates were obtained as described previously [24]. Subsequently, 100 µg of lysate was incubated with 8 µL DNase I (2000 U/mL, New England Biolabs; New England Biolabs GmbH, Frankfurt/Main, Germany). The remaining IP protocol was conducted as described above.

### 2.7. Immunofluorescence and Immunohistochemistry (IHC)

Immunofluorescence in PDAC cells was performed as described previously [6], but using 13% or 4.5% of paraformaldehyde and utilizing the following primary and secondary antibodies: Myctag (Cell Signaling, #2272S, 1:200; Cell Signaling Technology, Danvers, MA, USA), HA (Cell Signaling, #2367, 1:100; Cell Signaling Technology, Danvers, MA, USA), pSer21EZH2 (Bethyl IHC-00388, 1:100; Bethyl, Montgomery, AL, USA), ALEXA Fluor 568 donkey anti-mouse (Invitrogen #A10037, 1:500; Carlsbad, CA, USA), and ALEXA Fluor 488 donkey anti-rabbit (Invitrogen #A32790, 1:500; Carlsbad, CA, USA). To assess the HA-NFATc1/pSer21EZH2 colocalization coefficient, 1228 DAPI-stained nuclei were counted in eleven IF pictures to determine the number of nuclei positive for both HA-NFATc1 and pSer21EZH2. H&E staining and IHC were performed as previously described [6]. Primary antibodies were: EZH2 (mouse: Cell Signaling #5246, 1:100; human: Leica NCL-L-EZH2, 1:300; Leica Biosystems Wetzlar, Germany), NFATc1 (abcam ab25916, 1:200; Cambridge, UK), and pSer21EZH2 (Bethyl IHC-00388, 1:50; Bethyl, Montgomery, AL, USA). 

### 2.8. Chromatin Immunoprecipitation (ChIP)- and RNA-Sequencing Data Analysis 

ChIP analysis was performed as described previously [7]. Briefly, cells were fixed using 1% formaldehyde in PBS for 20 min before the reaction was quenched by adding 1.25 M glycine for 5 min. NFATc1- (Genetex, GTX22796 4 µg; Genetex, Irvine, CA, USA), H3K27ac- (Genetex, GTX128944), or rabbit IgG- (2 µg, Santa Cruz sc-2027) antibodies were added to precleared chromatin for overnight incubation. Finally, qRT-PCR was conducted with primers depicted in Supplementary Table S3. For joint analysis of publicly available genome-wide binding and transcriptome data we utilized the following previously described ChIP- and RNA-seq datasets: NFATc1 ChIP-seq [15], RNA-seq following NFATc1 knockdown (performed for 48 h) [19], EZH2 ChIP-seq (GSE153537), RNA-seq (in clones with stable EZH2 knockdown, GSE153491) [7]. For ChIP-seq, BigWig files were obtained from the mentioned references and were visualized using an integrative genomics viewer (IGV version 2.5.3). Differential binding (Diffbind) analysis was performed to identify the regions occupied by NFATc1 and EZH2 using the Bioconductor R package Diffbind run on R version 3.6.1. Further, Genomic Regions Enrichment of Annotations Tool (GREAT) analysis was used to identify associated genes with NFATc1 and EZH2 bound regions. For the analysis of RNA-seq data, genes with FPKM values <0.2 were excluded from the analysis to reduce background signals. Genes expressed with a log2 fold change of 0.5 and q value < 0.1 were considered significant. The pathway analysis was performed in EnrichR database (https://maayanlab.cloud/Enrichr/ accessed on 5 October 2021). The enrichment was computed based on Combined score and the top 10 pathways were plotted. *p* value < 0.05 were considered significant. Heatmap was plotted using pheatmap function in R (version 3.6.1). Venn diagrams were plotted in Venn (https://bioinformatics.psb.ugent.be/webtools/Venn/ accessed on 5 October 2021). 

### 2.9. Statistical Analysis

Two tailed student’s *t* test was used to calculate the column statistics. Data are represented as mean ± SD. and *p* value of <0.05 was considered as statistically significant. *p* values <0.05, <0.01, *p* < 0.001, and *p* < 0.0001 are depicted as *, **, *** and ****, respectively. For the pathway analysis, *p* values < 0.05 were considered significant. For the correlation plots, pearson correlation was used to calculate the correlation coefficient ‘r’ and determine the significance.

## 3. Results

### 3.1. NFATc1 and EZH2 Are Co-Expressed in a Subset of Murine and Human PDAC Samples

**Figure 1:**
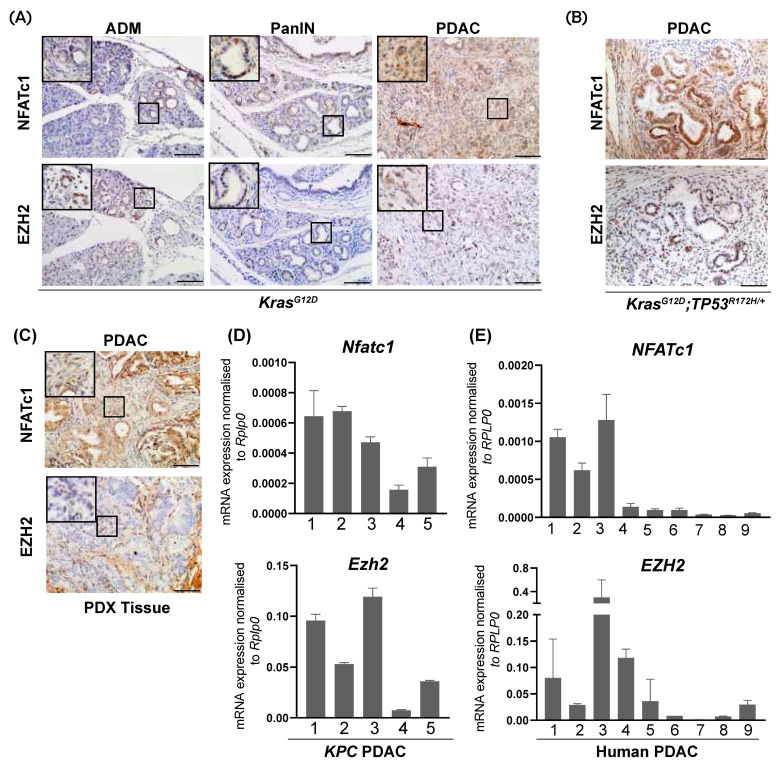
NFATc1 and EZH2 are co-expressed in a subset of murine and human PDAC samples. (**A**) Representative immunohistochemical analysis of NFATc1 and EZH2 in acinar cells, and pre-neoplastic lesions (ADM and PanIN, 6 mice each) and invasive PDAC in *Kras^G12D^* mice (*n* = 6 mice). Magnification 20×; Scale bar: 50 µm. (**B**) Representative immunohistochemical analysis of NFATc1 and EZH2 in PDAC of *KPC* mice (*n* = 6). Magnification 20×; Scale bar: 50 µm. (**C**) Representative immunohistochemical analysis of NFATc1 and EZH2 in a PDAC Patient-Derived Xenograft (PDX) model. Magnification 20×; Scale bar: 50 µm. (**D**,**E**) *NFATc1*- and *EZH2*- mRNA expression in *KPC* (**D**) and human PDAC (**E**) upon micro-dissection of tumor cells. Every bar represents one mouse (**D**) or patient (**E**). ADM: Acinar to Ductal Metaplasia, PanIN: Pancreatic Intraepithelial Neoplasia.

Utilizing a tissue microarray of 107 human PDAC samples, we previously detected abundant EZH2 expression in 70% of those human PDAC specimen characterized by high epithelial nuclear NFATc1 expression [14]. In order to explore the existence of an NFATc1-EZH2 axis in pancreatic carcinogenesis and PDAC progression, we took advantage of well-characterized transgenic mouse models of PDAC development and progression (*Kras^G12D^-* and *Kras^G12D^;TP53^R172H/+^-* (*KPC*) mice [5,20]) and explored NFATc1- and EZH2 expression in epithelial pancreatic cells. As described previously [5], *Kras^G12D^* mice display all stages of PDAC development including acinar-to-ductal metaplasia (ADM) and Pancreatic Intraepithelial Neoplasia (PanIN)—lesions which eventually progress to PDAC in advanced age. While comparably low nuclear NFATc1 and EZH2 expression were detectable in pancreatic acinar cells, immunohistochemical analyses exposed a robust increase of NFATc1 and EZH2 expression in ADM- and PanIN-positive *Kras^G12D^* mice with maximal expression occurring in PDAC (Figure 1A). Accordingly, concomitant expression of NFATc1 and EZH2 was also detected in invasive pancreatic tumors of the fast-progressing *KPC* model [20] (Figure 1B) and in a PDAC Patient-Derived Xenograft (PDX) model (Figure 1C). To explore, whether a co-expression of both oncogenic drivers already occurs at the level of gene transcription, we isolated mRNA from murine (*KPC*) and human PDAC. Given the cellular heterogeneity of these tumors, exploration of mRNA-expression was restricted to epithelial tumor parts obtained by manual microdissection. Interestingly, we found that high *Nfatc1* expression in murine PDAC showed a trend towards higher *Ezh2* levels (Figure 1D, Supplementary Figure S1A, r = 0.63) and, that the extent of *NFATc1*- and *EZH2* mRNA expression significantly correlated with each other in human PDAC (Figure 1E, Supplementary Figure S1B, r = 0.75). Together, these findings indicate a co-expression of NFATc1 and EZH2 in a subset of the PDAC specimen.

### 3.2. NFATc1 Induces EZH2 Expression at the Level of Gene Transcription

**Figure 2:**
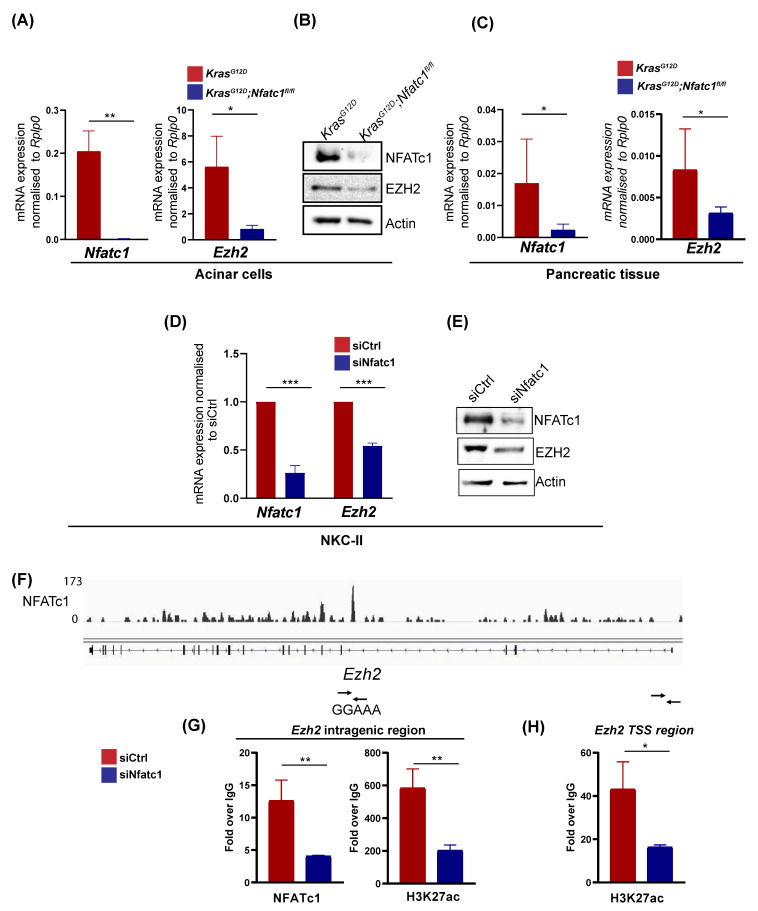
NFATc1 induces *EZH2* expression at the level of gene transcription. (**A**) *Nfatc1* (left) and *Ezh2* (right) mRNA expression in acinar cells isolated from *Kras^G12D^* and *Kras^G12D^;Nfatc1^fl/fl^* mice (*n* = 3 mice/genotype). Values represent mean +/− SD (two-tailed unpaired student’s *t*-test). (**B**) NFATc1 and EZH2 expression in whole cell lysates obtained from acinar cells isolated from 6-week old *Kras^G12D^* and *Kras^G12D^;Nfatc1^fl/fl^* mice. (**C**) Pancreatic tissue isolated from 12-week old *Kras^G12D^* and *Kras^G12D^;Nfatc1^fl/fl^* mice (*n* = 3 mice/genotype) was subjected to *Nfatc1* (left) and *Ezh2* (right) mRNA expression analysis. Values represent mean +/− SD (two-tailed unpaired student’s *t*-test). (**D**) NKC-II cells were subjected to NFATc1 knockdown and explored regarding *Nfatc1*- and *Ezh2* expression via qRT-PCR. Values represent mean +/− SD from 3 independent experiments (two-tailed unpaired student’s *t*-test). (**E**) Western Blot analysis in NKC-II cells upon siRNA-mediated NFATc1 knockdown. (**F**) IGV profile of NFATc1 ChIP-seq analysis [15] suggesting NFATc1 binding at an intragenic region of the *Ezh2* gene which harbors the NFAT consensus site GGAAA. (**G**) NFATc1 and H3K27ac ChIP analysis in NKC-II cells in the presence and absence of NFATc1 at the intragenic region for which ChIP-seq analysis suggested NFATc1 binding. (**H**) H3K27ac occupancy at the *Ezh2* TSS region was assessed by ChIP analysis in the presence and absence of NFATc1. (**G**,**H**) Percent of input was determined for IgG-, NFATc1- and H3K27ac binding and subsequently, NFATc1- and H3K27ac binding were normalized to IgG. Values represent mean +/− SD from *n* = 3 (two-tailed unpaired student’s *t*-test). Data are represented as mean ± SD. and *p* value of <0.05 was considered as statistically significant. *p* values < 0.05, < 0.01, *p* < 0.001, and *p* < 0.0001 are depicted as *, **, and ***, respectively.

We have previously shown that in the context of oncogenic KRAS, EZH2 induces *NFATc1* expression by targeting the *NFATc1* promoter for transcriptional activation [14]. Here we asked, whether NFATc1 is comparably sufficient to induce *EZH2* transcription. To address this hypothesis, we took advantage of *Kras^G12D^;Nfatc1^fl/fl^* mice with a pancreas-specific depletion of the *Nfatc1* gene [15,16] and performed *Ezh2* mRNA expression analyses at different steps of pancreatic carcinogenesis. Compared to *Kras^G12D^* mice, *Ezh2* mRNA and protein expression was strongly abolished in acinar cells extracted from *Kras^G12D^;Nfatc1^fl/fl^* mice (Figure 2A,B, Supplementary Figure S1C). Consistently, reduced *Ezh2* expression persisted in *Nfatc1*-deficient mice analyzed at the age of 12 weeks, where pre-neoplastic pancreatic lesions are evident (Figure 2C) [16]. To explore, whether NFATc1-dependent EZH2 induction is also evident in established PDAC, we utilized PDAC cells isolated from tumor-bearing transgenic mice characterized by constitutive nuclear NFATc1 expression and oncogenic Kras activation (*cnNFATc1;Kras^G12D^* mice, NKC-II cells [15]) and subjected these cells to siRNA-mediated NFATc1 knockdown. In line with previous genome-wide expression analysis conducted in the presence and absence of NFATc1 [15], NFATc1-depletion resulted in a strong reduction of *EZH2* expression on both the mRNA and protein level (Figure 2D,E, Supplementary Figure S1D). Given, that NFATc1 ChIP-seq analysis previously performed in NKC-II cells [15] revealed NFATc1 binding at the intragenic region of *Ezh2* (Figure 2F), we proposed that the TF directly targets the methyltransferase to control its transcription. Indeed, independent NFATc1 ChIP analysis confirmed NFATc1 binding at the intragenic region of the *Ezh2* gene (Figure 2G). In accordance with the NFATc1-dependent regulation of *Ezh2* transcription (Figure 2A,C,D), the knockdown of NFATc1 significantly reduced the occupancy of H3K27ac, a histone mark indicative for the activity of enhancer regions and Transcriptional Start Sites (TSS) [25], both at the intragenic (Figure 2G) as well as at the TSS region (Figure 2H) of the *Ezh2* gene. These findings suggest that NFATc1 directly targets the *Ezh2* gene for transcriptional activation. Together, our findings suggest NFATc1 as a transcriptional regulator of *EZH2* expression in PDAC development and progression.

### 3.3. NFATc1 Is Involved in the Regulation of a Subset of EZH2-Dependent Gene Signatures

**Figure 3:**
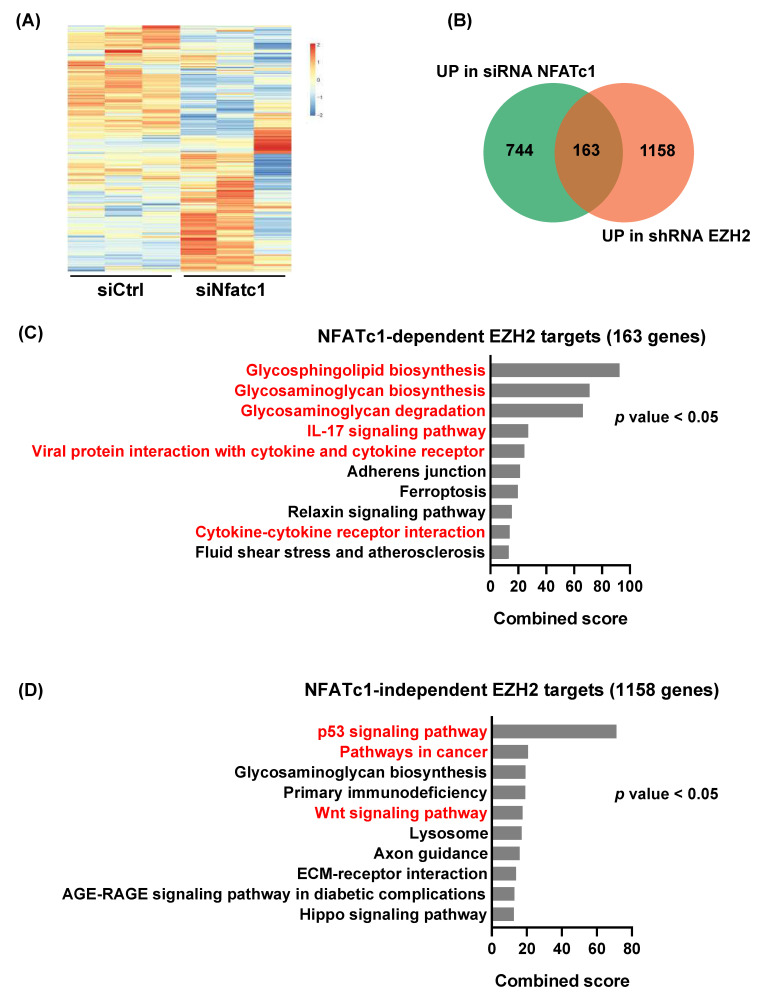
NFATc1 is involved in the regulation of a subset of EZH2-dependent gene signatures. (**A**) Heatmap illustrating NFATc1-dependent regulation of the subset of genes upregulated upon EZH2 knockdown as identified upon RNA-seq analysis in NKC-II cells (log2FC > 0.5, q value < 0.1, EZH2-repressed genes, *n* = 1321 genes) [7,19]. (**B**) A Venn diagram overlaying NFATc1- and EZH2-target genes found to be upregulated upon NFATc1- and EZH2-knockdown, respectively (log2FC > 0.5, q value < 0.1). The 163 overlapping target genes are considered as *NFATc1*-dependent *EZH2* targets. The 1158 genes upregulated only upon EZH2 knockdown are further referred to as *NFATc1*-independent *EZH2* target genes. (**C**,**D**) Pathway analysis of *NFATc1*-dependent (**C**) and *NFATc1-independent* (**D**) *EZH2* target genes using *EnrichR.* Enrichment was estimated based on combined score and the top 10 pathways were plotted. Distinct pathways in the groups of NFATc1-dependent and independent EZH2 targets are highlighted in red. *p* value < 0.05 were considered significant.

Based on our findings suggesting an NFATc1-EZH2 axis operative in PDAC, we next aimed at exploring the impact of NFATc1 expression on EZH2-dependent gene regulation. To this end, we integrated RNA-seq data obtained from EZH2 positive (shRNA control) and negative (shRNA EZH2) NKC-II cells [7] with NKC-II transcriptome data generated in the presence and absence of NFATc1 (siRNA control vs. siRNA NFATc1 [19]). A reduction of *Ezh2* expression following NFATc1 knockdown was confirmed in all replicates utilized for RNA-seq analysis (Supplementary Figure S1E). In order to dissect, whether NFATc1 impacts EZH2-dependent gene regulation, we first filtered for genes which were found to be upregulated upon EZH2 knockdown (EZH2 repressed genes, *n* = 1321 genes) and subsequently explored the consequences of NFATc1 knockdown on the expression of these genes (Figure 3A). Upon exclusion of low expressed genes (FPKM < 0.1), the majority of genes upregulated upon EZH2-knockdown remained unchanged or were downregulated upon NFATc1 loss (*n* = 1158 genes, further considered as NFATc1-independent EZH2 target genes). We also identified a cluster of 163 EZH2-repressed genes which were significantly upregulated upon NFATc1 knockdown (12%, considered as NFATc1-dependent EZH2 target genes, Figure 3B). Pathway analysis linked these 163 genes predominantly to glycosphingolipid and glycosaminoglycan metabolism and cytokine regulation (Figure 3C). Interestingly, these pathways significantly differed from the signatures identified for the NFATc1-independent EZH2 target genes, which associated with p53- and Wnt-signaling (Figure 3D). Together, these findings imply that NFATc1 might be critically involved in at least indirectly controlling a subset of EZH2-dependent gene repression programs in PDAC cells, but also illustrate that the vast majority of EZH2-dependent gene regulation occurs in an NFATc1-independent manner.

### 3.4. NFATc1 and EZH2 Biochemically Interact with Each Other but Are Not Involved in Joint Regulation of Direct Target Gene Transcription

**Figure 4:**
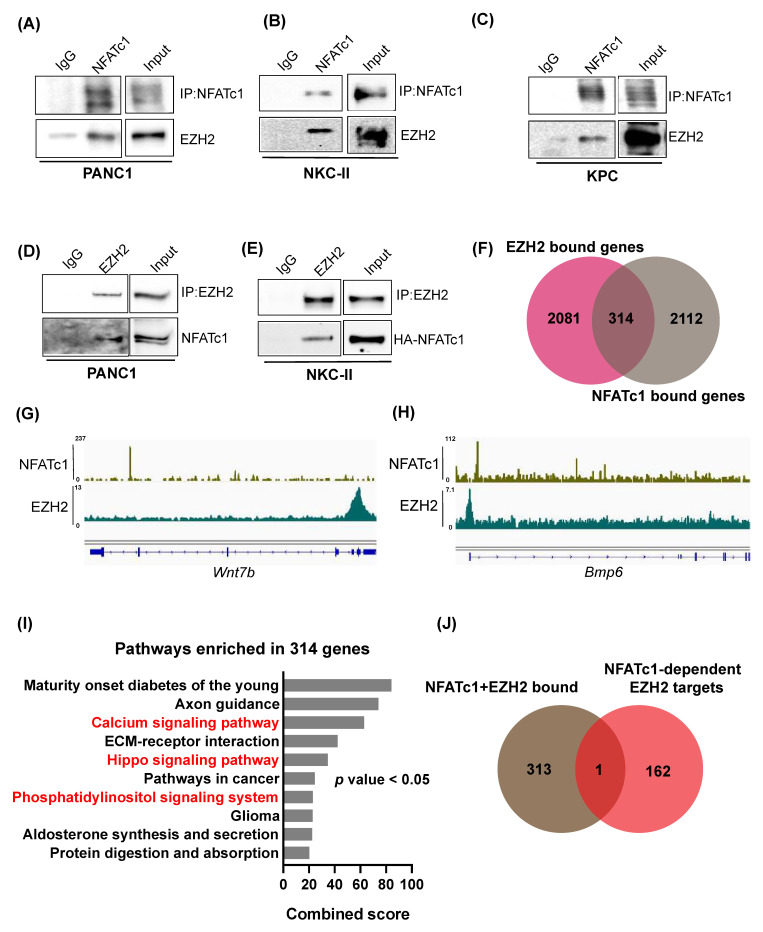
NFATc1 and EZH2 biochemically interact with each other, but are not involved in joint regulation of direct target gene transcription. (**A**–**E**) Immunoprecipitation of endogenous NFATc1 (**A**–**C**) and endogenous EZH2 (**D**,**E**) in the indicated cells lines was followed by Western Blot analysis. IP studies in A–C were performed in whole cell lysates, while nuclear lysates were utilized for IPs depicted in D,E. (**F**) Overlay of target genes bound by NFATc1 and EZH2 as identified by ChIP-seq analysis [7,15] in NKC-II cells. (**G**,**H**) IGV profiles demonstrating NFATc1 and EZH2 occupancy on the *Wnt7b* (**G**) and *Bmp6* (**H**) genes in NKC-II cells. (**I**) Pathways (as identified by EnrichR) enriched in the 314 NFATc1- and EZH2-bound genes. Cancer-related pathways are highlighted in red. Pathways were sorted based on combined score and the top 10 pathways were plotted. *p* values < 0.05 were considered significant. (**J**) Overlay of NFATc1- and EZH2 bound genes with the 163 *NFATc1*-dependent EZH2 target genes (as shown in Figure 3B).

NFAT proteins are characterized by a very low DNA binding affinity [26]. In order to sufficiently control gene transcription, the transcription factor forms protein complexes with additional transcriptional regulators including other transcription factors [15,16,18,26] or chromatin regulatory proteins [6,17]. Interestingly, genes encoding for NFAT partner proteins in PDAC regularly display direct NFATc1 targets, as e.g., illustrated for Sox2 [18] and STAT3 [15]. Consequently, we asked, whether NFATc1-dependent induction of *EZH2* transcription is followed by NFATc1:EZH2 complex formation. Co-immunofluorescence studies in NKC-II cells revealed a co-localization of both proteins in PDAC cells (Supplementary Figure S2A). Moreover, upon immunoprecipitation of endogenous NFATc1 or EZH2, we observed robust NFATc1:EZH2 complex formation in all tested human and murine PDAC cells (Figure 4A–E, Supplementary Figure S2B). Given, that NFAT partner proteins do not only increase NFAT DNA binding affinity, but further critically impact target gene selection as well as the mode of transcriptional regulation [26], we next asked whether the NFATc1:EZH2 complex jointly regulates gene expression by binding a subset of joint target genes. Integration of EZH2-[7] and NFATc1-[15] ChIP-seq studies performed in NKC-II cells revealed 314 genes co-occupied by both factors (Figure 4F). Exemplary tracks of the NFATc1- and EZH2 ChIP-seqs are shown in Figure 4G,H. Interestingly, NFATc1- and EZH2 co-occupied genes were involved in pathways with critical implications in cancer, such as calcium or Hippo signaling (Figure 4I). However, only a single NFATc1 and EZH2 co-occupied target gene overlapped with the 163 genes which were identified to be up-regulated by both, NFATc1- and EZH2-knockdown (*Bmp6*, Figure 4J). These data strongly argue against a model linking the NFATc1:EZH2 complex to joint direct target gene repression. Given, that NFATc1-dependent gene regulation has been predominantly linked to activation of gene transcription [15] and based on previous findings suggesting the existence of repression-independent EZH2 activities [14,21,27], we also explored, whether the 314 NFATc1/EZH2-co-occupied target genes are jointly induced by the protein complex. Consistent with our findings shown in Figure 4J, these analyses revealed only one overlapping gene (*Epcam*, Supplementary Figure S2C). Together, these findings suggest that the NFATc1:EZH2 complex is most likely not involved in the transcriptional regulation of gene expression.

### 3.5. NFATc1:EZH2 Complex Formation Occurs in a Chromatin-Independent Manner and Requires Posttranslational EZH2 Phosphorylation

**Figure 5:**
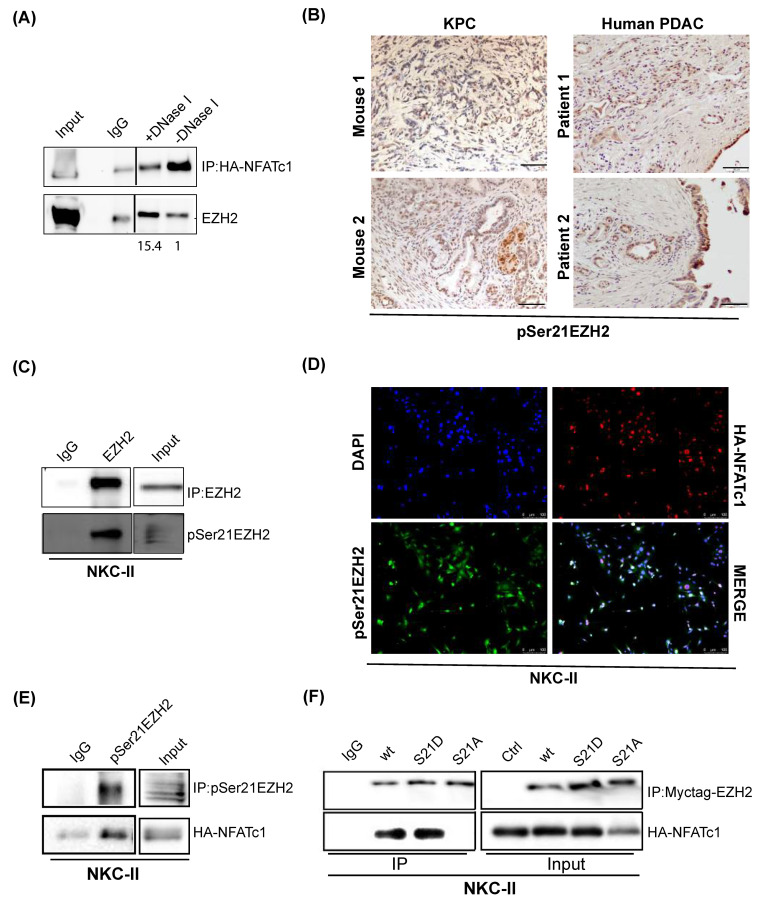
NFATc1:EZH2 complex formation occurs in a chromatin-independent manner and requires posttranslational EZH2 phosphorylation. (**A**) IP of HA-tagged NFATc1 in nuclear NKC-II cell lysates in the presence and absence of DNase I. EZH2 band intensities were normalized to the respective NFATc1 bait protein band intensities using image J (version 1.50b). Subsequently, the band intensity quotient of the DNase I-treated sample was normalized to the quotient determined for the DNase I-negative sample and is illustrated under the Western Blot. IgG was used as control. Lines between IgG and DNAse I samples indicate that the blot has been cut. (**B**) Immunohistochemical analysis of pSer21EZH2 in *KPC* (left, representative for *n* = 6 mice) and human (right) PDAC (representative for 3 patients). Magnification 20×; Scale bar: 50 µm. (**C**) IP samples of endogenous EZH2 were stained for EZH2 and pSer21EZH2 to confirm the specificity of the antibody. (**D**) Co-immunofluorescence of pSer21EZH2 and HA-NFATc1 in NKC-II cells. Twenty-five percent of nuclei (308/1228) harbor co-localization. Scale bar: 100 µm. (**E**) Western Blot analysis upon IP of pSer21EZH2 in NKC-II cells. (**F**) IP of Myctag-EZH2 upon overexpression of EZH2 wildtype (EZH2 wt), a phosphomimicking- (EZH2 S21D), and a non-phosphorylatable- (EZH2 S21A) construct in NKC-II cells. Input samples were loaded to confirm successful overexpression of the EZH2 constructs.

Given, that our findings did not indicate a critical involvement of the NFATc1:EZH2 complex in joint regulation of target gene transcription, we asked whether the protein complex might be involved in chromatin-independent functions. In line with this hypothesis, IP conducted in the presence of the DNA fragmentation-inducing enzyme DNase I revealed an enhancement of NFATc1:EZH2 complex formation upon disrupting DNA integrity (Figure 5A). Given, that chromatin-independent EZH2 functions evident in glioblastoma [27] and prostate cancer [21] have been linked to posttranslational phosphorylation of the serine 21 residue of the methyltransferase (pSer21EZh2) [21,27], we explored the impact of this posttranslational EZH2 modification on NFATc1-EZH2 co-expression and NFATc1:EZH2 complex formation. Importantly, we detected pSer21EZH2 expression in preneoplastic pancreatic lesions (Supplementary Figure S3), in murine and human PDAC tissue (Figure 5B) and in primary murine PDAC cells (Figure 5C). Co-immunofluorescence experiments utilizing an antibody specific for the detection of serine 21 phosphorylated EZH2 (pSer21EZH2) [27] revealed a co-localization with NFATc1 in PDAC cells in 25% (308/1228) of counted nuclei (Figure 5D). Moreover, IP experiments showed robust complex formation of the transcription factor and endogenous pSer21EZH2 in NKC-II cells (Figure 5E). In order to explore, whether posttranslational EZH2 phosphorylation is required for NFATc1:EZH2 complex formation, we overexpressed either wildtype EZH2 (EZH2 wt), or EZH2 constructs harboring a constitutive phosphorylation of the serine 21 residue (EZH2 S21D) or a phosphorylation-dead mutant (EZH2 S21A) in NKC-II cells and performed IP of Myctag-EZH2. While these studies confirmed NFATc1 complex formation with wildtype EZH2 and revealed a comparably strong interaction with EZH2 S21D, we did not detect NFATc1 binding upon precipitation of non-phosphorylatable S21A EZH2 (Figure 5F), suggesting that the posttranslational EZH2 phosphorylation on serine 21 indeed constitutes a prerequisite for the physical interaction of NFATc1 and EZH2.

In summary, we show that NFATc1 targets the *Ezh2* gene for transcriptional activation and subsequently forms a robust complex with the histone methyltransferase in PDAC. Despite the significance of both factors in individually controlling tumor promoting gene signatures in PDAC development and progression [7,15,16,19], our findings suggest a chromatin-independent assembly of both proteins restrictively upon posttranslational EZH2 phosphorylation, thus pointing towards chromatin-independent implications of the NFATc1:EZH2 complex in PDAC development and progression. 

## 4. Discussion

EZH2 deregulation represents a frequent event in cancer and has been detected in a plethora of tumor entities [12,28,29,30,31,32,33,34,35]. While dysregulation of EZH2 activity in hematological malignancies is predominantly caused by activating mutations [36,37], abundance of the methyltransferase in solid cancers including PDAC is mainly linked to overexpression [7,12,28,29,30,31,32]. A variety of signaling pathways and transcriptional mechanisms have been linked to EZH2 overexpression in cancer. The pRb-E2F signaling pathway, for instance, induces EZH2 expression in several tumor entities [38,39,40,41]. Upon phosphorylation-induced release of E2F from the pRb-E2F complex, the activated transcription factor E2F binds the *EZH2* promoter for subsequent activation of gene transcription [38,39], thus resulting in a highly dynamic regulation of EZH2 expression throughout the cell cycle. In addition to E2F, several transcription factors with critical involvement in carcinogenesis and tumor progression control *EZH2* transcription, such as MYC, NF-YA, STAT3, ETS, and ELK1 [42,43,44,45,46]. With NFATc1, we identified a previously unknown inducer of EZH2 expression in PDAC development and progression and demonstrated that the tumor-promoting transcription factor directly targets the *EZH2* gene for transcriptional activation. It is noteworthy, that some of the transcription factors which have been previously linked to *EZH2* activation, albeit in other cancer entities, underlie transcriptional control by NFATc1 (MYC [17,24], STAT3 [15]) or represent well-characterized NFATc1 partner proteins (STAT3 [15], ELK1 [17]). Hence, NFATc1-dependent regulation of *EZH2* expression is not unlikely to occur in concert with one of those transcription factors. Our observations demonstrating: (i) NFATc1 binding at an intragenic region of the *Ezh2* gene and (ii) the NFATc1-dependent regulation of H3K27ac occupancy at this site suggest that NFATc1 targets a putative *Ezh2* enhancer which, once activated, increases the transcriptional activation of *Ezh2*. Although this assumption requires further experimental validation, this finding is in accordance with previous results indicating that NFATc1-dependent target gene regulation predominantly occurs at enhancer, rather than promoter/TSS genomic regions [15]. 

In contrast to all previously identified NFATc1 partner protein complexes with critical involvement in PDAC development and progression [15,16,17,18,26], our findings do not link the NFATc1:EZH2 complex to regulation of joint transcriptional processes. Although our ChIP-seq analyses revealed a partial overlap of EZH2- and NFATc1 occupancies, we were not able to identify direct NFATc1-/EZH2-bound gene signatures, which were jointly either activated or repressed by the NFATc1:EZH2 complex. These data suggest that both proteins might bind these target genes only individually and in a highly dynamic and temporally distinct manner. Instead of joint gene regulation, our interaction studies in the absence of intact DNA link the NFATc1:EZH2 complex to chromatin-independent activities. Although overexpression of EZH2 typically associates with increased PRC2 recruitment to chromatin, abundance of the H3K27me3 mark and gene silencing [7], some studies point towards a critical involvement of EZH2 activity beyond transcriptional repression [14,21,27]. For instance, in breast cancer, EZH2 interacts with β-catenin or members of the NF-κB transcription factor family in estrogen receptor-positive or -negative tumor subtypes, respectively. In both cases, EZH2 activity is associated with activation of gene transcription [47,48]. In colon cancer, EZH2 biochemically associates with β-catenin and the PCNA-associated factor PAF to induce the expression of Wnt target genes [49]. While EZH2-dependent transcriptional activation in colon cancer arises independently of the protein’s methyltransferase activity [49], the activating transcriptional properties of EZH2 in castration-resistant prostate cancer rely on its methyltransferase activity, but do not occur in concert with the other PRC2 components [21]. Whether the EZH2 methyltransferase activity and/or other PRC2 proteins are required for NFATc1:EZH2 complex formation remains elusive. However, we characterized the posttranslational EZH2 serine 21 phosphorylation as a prerequisite for NFATc1:EZH2 complex formation. Consistent with these findings, phosphorylation of the serine 21 EZH2 residue has been shown to be required for the interaction of EZH2 with other transcriptional regulators, such as the androgen receptor and STAT3 in prostate cancer [21] and glioblastoma [27], respectively. AKT was identified as the kinase responsible for serine 21 phosphorylation of EZH2 [21,27]. Interestingly, we did not observe a reduction of pSer21EZH2 expression upon interfering with AKT activity in PDAC cells (data not shown), suggesting the involvement of alternative kinases. Besides AKT, a plethora of enzymes have been associated with posttranslational EZH2 phosphorylation at a variety of amino acid residues and in different cancer entities [50]. Interestingly, one of the proteins involved in posttranslational EZH2 modification is the glycogen synthase kinase 3β (GSK3β) [51]. GSK3β does not only act as an important driver of PDAC development, progression, and resistance [52,53,54], it also represents a well-characterized regulator of NFAT protein stability and fosters NFAT interaction with in PDAC [52]. Although GSK3β-dependent posttranslational EZH2 phosphorylation has hitherto exclusively been linked to the serine 363 residue [51], the kinase might be involved in phosphorylation of additional EZH2 protein sites. Given the implications of GSK3β in: (i) stabilizing NFAT proteins and (ii) phosphorylating EZH2, and considering our findings pointing towards chromatin-independent functions of the NFATc1:EZH2 complex, we speculate that the Ser21EZH2 phosphorylation might contribute to GSK3β-controlled NFAT stability in PDAC progression.

Together, our findings describe a previously unappreciated NFATc1-EZH2 axis in PDAC and indicate that chromatin-independent EZH2 activities might represent a critical component of the oncogenic properties of the methyltransferase in PDAC. By identifying the posttranslational serine 21 EZH2 phosphorylation as a prerequisite for stable complex formation with NFATc1, our data set stage for future studies aiming at the identification of the involved upstream kinases and the mechanistic and tumor-biological implications of the NFATc1:EZH2 complex in pancreatic pathogenesis.

## Supplementary Materials

The following are available online at https://www.mdpi.com/article/10.3390/cells10123463/s1, Figure S1: (A,B) Correlation plots of NFATc1- and EZH2 mRNA expression in *KPC* (A) and human (B) PDAC (corresponding to Figure 1D,E). (C,D) Densitometry of NFATc1 and EZH2 expression upon NFATc1 knockdown (referring to Western Blot analysis depicted in Figure 2B and E, respectively); (E) FPKM values of *Ezh2* as identified in the triplicates of siRNA control (siCtrl) and siRNA NFATc1 (siNfatc1) RNA-seq samples [19]. Figure S2: (A) Co-Immunofluorescence of HA-NFATc1 and Myctag-EZH2 upon overexpression of EZH2 wt in NKC-II cells. (B) Nuclear lysates obtained from NKC-II cells were subjected to HA-NFATc1 IP. Western Blot analysis confirms interaction with EZH2. (C) Overlay of NFATc1- and EZH2 bound genes with the 103 genes down-regulated upon NFATc1- and EZH2 knockdown (log2FC > 0.5, q value < 0.1); Figure S3: Immunohistochemical analysis of pSerEZH2 expression in 4- and 12-week old *Kras^G12D^* mice harboring normal pancreatic tissue or ADM and PanIN lesions. Representative pictures of 3 mice/age. Scale bar: 100 µm; Table S1: qPCR primer sequences; Table S2: Primary and secondary antibodies utilized for Western Blot analysis; Table S3: ChIP primer sequences.
