# Supplementary material for: Chromatin-Independent Interplay of NFATc1 and EZH2 in Pancreatic Cancer
Source: Cells. 2021 Dec 8;10(12):3463. doi: 10.3390/cells10123463 (PMC8700089; doi:10.3390/cells10123463)
Supplement: Supplementary file 1 [file cells-10-03463-s001.zip › cells-1466158-supplementary.pdf]

cells MDPI

Supplementary Materials

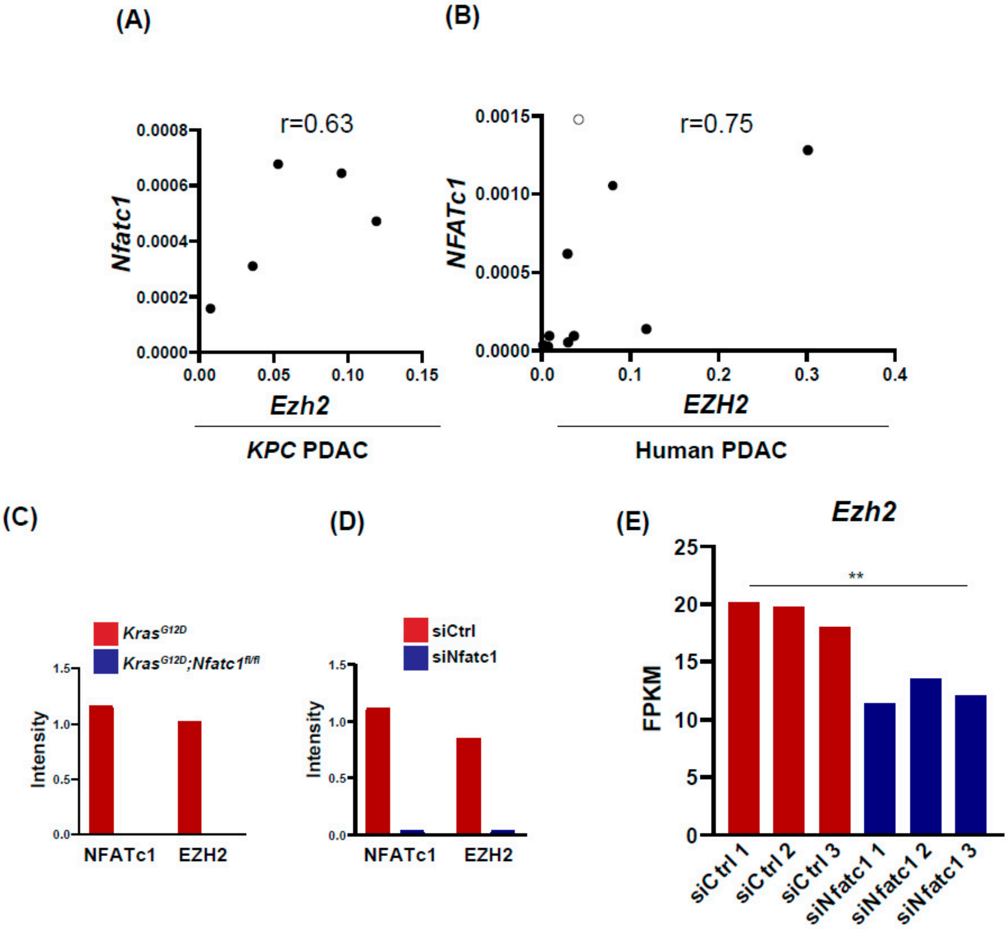

Figure S1. (**A**,**B**) Correlation plots of *NFATc1*- and *EZH2* mRNA expression in *KPC* (**A**) and human (**B**) PDAC (corresponding to Figures 1D,E). The pearson correlation coefficient ´r´ is depicted in the figures. (**C**,**D**) Densitometry of NFATc1 and EZH2 expression upon NFATc1 knockdown (referring to Western Blot analysis depicted in Figure 2B and E, respectively). The densitometry is representative for 3 biological replicates. (**E**) FPKM values of *Ezh2* as identified in the triplicates of siRNA control (siCtrl) and siRNA NFATc1 (siNfatc1) RNA-seq samples [19].

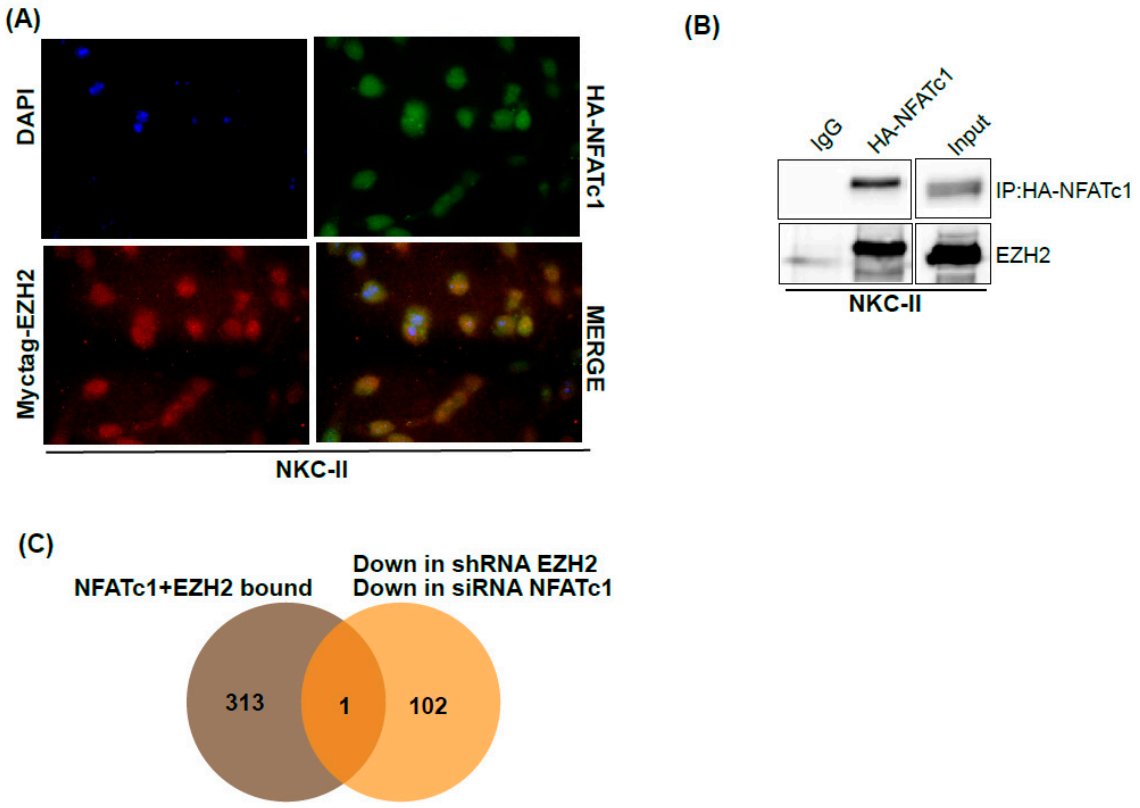

Figure S2. (**A**) Co-Immunofluorescence of HA-NFATc1 and Myctag-EZH2 upon overexpression of EZH2 wt in NKC-II cells. (**B**) Nuclear lysates obtained from NKC-II cells were subjected to HA-NFATc1 IP. Western Blot analysis confirms interaction with EZH2. (**C**) Overlay of NFATc1- and EZH2 bound genes with the 103 genes down-regulated upon NFATc1- and EZH2 knockdown (log2FC > 0.5, q value < 0.1).

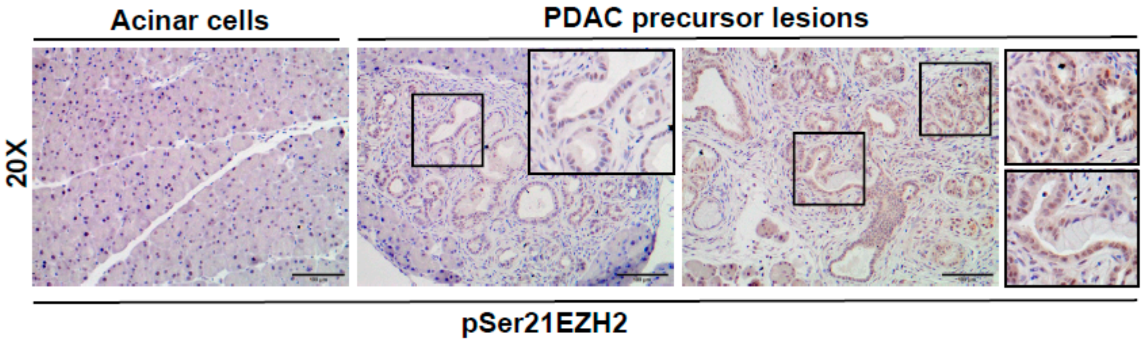

Figure S3. Immunohistochemical analysis of pSerEZH2 expression in 4- and 12-week old *Kras*^{G12D} mice harboring normal pancreatic tissue or ADM and PanIN lesions. Representative pictures of 3 mice/age. Scale bar: 100 μm.

Table S1. qPCR primer sequences.

Gene	Sequence	Species
Ezh2 FP	CAACCCGAAAGGGCAACAAA	Mouse
Ezh2 RP	ACCAGTCTGGATAGCCCTCT	Mouse
Nfatc1 FP	GCCTTTTGCGAGCAGTAT	Mouse
Nfatc1 RP	GCTGCCTTCCGTCTCATA	Mouse
RPLP0 FP	TGGGCAAGAACACCATGATG	Mouse/Human
RPLP0 RP	AGTTTCTCCAGAGCTGGGTTGT	Mouse/Human
EZH2 FP	AAAGAACTCACCGAACAGCA	Human
EZH2 RP	CAGAAAAGCGTATGAAAGGAGTG	Human
NFATC1 FP	GTTCCTACCCCACAGTCATTCAG	Human
NFATC1 RP	CCCGCAGGTAATACTTCCTTTTG	Human

Table S2. Primary and secondary antibodies utilized for Western Blot analysis.

Antibody	Company	Dilution
EZH2	Cell Signaling #5246	1:1000
pSer21EZH2	Bethyl IHC-00388	1:1000
NFATc1	Santa Cruz sc-7294	1:500
HA	Cell Signaling #2367	1:1000
Myctag	Cell Signaling #2272S	1:1000
ACTIN-HRP	Sigma	1:40000
Anti-rabbit (IgG) HRP	Cell Signaling #7076	1:10000
Anti-mouse (IgG) HRP	Cell Signaling #7074	1:10000

Table S3. ChIP-seq primer sequences.

Gene	Sequence
Ezh2 TSS *FP*	TCTGTCTCACAACTGAGGCACT
Ezh2 TSS RP	TGTGGTAAGAAGGCATTATGTCA
Ezh2 intragenic *FP*	ATGCCTTCTTACCACAGTGACTT
Ezh2 intragenic RP	CAGGGCACGAATTTTTACCACAT
